# Vacuum-Assisted MonoTrap^TM^ Extraction for Volatile Organic Compounds (VOCs) Profiling from Hot Mix Asphalt

**DOI:** 10.3390/molecules29204943

**Published:** 2024-10-18

**Authors:** Stefano Dugheri, Giovanni Cappelli, Niccolò Fanfani, Donato Squillaci, Ilaria Rapi, Lorenzo Venturini, Chiara Vita, Riccardo Gori, Piero Sirini, Domenico Cipriano, Mieczyslaw Sajewicz, Nicola Mucci

**Affiliations:** 1 Department of Experimental and Clinical Medicine, University of Florence, 50134 Florence, Italy; giovanni.cappelli@unifi.it (G.C.); niccolo.fanfani@unifi.it (N.F.); donato.squillaci@unifi.it (D.S.); ilaria.rapi@edu.unifi.it (I.R.); lorenzo.venturini@unifi.it (L.V.); nicola.mucci@unifi.it (N.M.); 2 PIN—University Center “Città di Prato” Educational and Scientific Service, University of Florence, 59100 Prato, Italy; chiara.vita@pin.unifi.it; 3 Department of Civil and Environmental Engineering, University of Florence, 50139 Florence, Italy; riccardo.gori@unifi.it (R.G.); piero.sirini@unifi.it (P.S.); 4 Ricerca sul Sistema Energetico (RSE), 20134 Milan, Italy; domenico.cipriano@rse-web.it; 5 Institute of Chemistry, University of Silesia, 40006 Katowice, Poland; mieczyslaw.sajewicz@us.edu.pl

**Keywords:** hot mix asphalt, monolithic material sorptive extraction, under vacuum extraction, volatile organic compounds, odor emission, gas chromatography-mass spectrometry

## Abstract

MonoTrap^TM^ was introduced in 2009 as a novel miniaturized configuration for sorptive sampling. The method for the characterization of volatile organic compound (VOC) emission profiles from hot mix asphalt (HMA) consisted of a two-step procedure: the analytes, initially adsorbed into the coating in no vacuum- or vacuum-assistance mode, were then analyzed following an automated thermal desorption (TD) step. We took advantage of the theoretical formulation to reach some conclusions on the relationship between the physical characteristics of the monolithic material and uptake rates. A total of 35 odor-active volatile compounds, determined by gas chromatography-mass spectrometry/olfactometry analysis, contributed as key odor compounds for HMA, consisting mainly of aldehydes, alcohols, and ketones. Chemometric analysis revealed that MonoTrap^TM^ RGC18-TD was the better coating in terms of peak area and equilibrium time. A comparison of performance showed that Vac/no-Vac ratios increased, about an order of magnitude, as the boiling point of target analytes increased. The innovative hybrid adsorbent of silica and graphite carbon monolith technology, having a large surface area bonded with octadecylsilane, showed effective adsorption capability, especially to polar compounds.

## 1. Introduction

Anthropic emissions of odorous compounds can strongly limit the use of the territory [1]. Therefore, the attempt to link the emissions of pollutants in the atmosphere, not only with concentration limits but also with limits for the odor impact, arises from the need to ensure that activities with significant osmogenic flows do not hinder the usability of the territory. Volatile chemicals are emitted from a variety of sources and, in recent years, the attention toward volatile organic compounds (VOCs) has increased, due to the consistent number of anthropogenic processes emitting them, for their environmental and health impact. In addition, VOC emissions can produce odor annoyance, which can reduce life quality [2].

Asphalt mixture plants represent common sources of odor-active VOCs, due to the high temperature (ranging from 150 to 180 °C) of the final hot mix asphalt (HMA) [3]. The characterization of odor impact from these plants is challenging; some phases of their production processes involve large and open areas, and transient emissions are common. Moreover, odors can be released from different plant areas, resulting in a complex source localization and a difficult estimation and measure of odors.

Generally, VOCs are defined based on their physical–chemical properties [4]. For Wang et al. [5], they are compounds with a boiling point (BP) below 100 °C at 101 kPa and vapor pressure (VP) higher than 13.3 Pa at 25 °C. In another definition provided by the European Union solvents directive (1999/13/EC) [6], VOCs are compounds with a VP of at least 10 Pa at 20 °C. Other example volatility criteria proposed by the United States (US) Environmental Protection Agency [7] and New Jersey Department of Environmental Protection [8] define volatile chemicals based on a VP greater than 133 Pa at 25 °C. Health Canada [9] classifies VOCs as compounds that have a BP roughly in the range of 50 to 250 °C.

The presence of odor-related VOCs in ambient air can result in the discomfort of the plant’s employees and the residents of neighboring areas. Despite the odor threshold (OT) of VOCs, the perception of an unpleasant odor is related to their concentration and relative hedonic tone; however, their influence on the perception is specific for each compound [10]. In addition, the understanding of these factors may also explain why, despite the enormous effort invested in creating odor parameters, governing bodies have had difficulty establishing fair and effective regulations that address community needs. European countries, like Portugal, Greece, and Austria, currently have no specific odor legislation, while other countries, like Germany, only present regulations for waste management activities.

The Italian Government gave its regions the power to regulate an odor impact [11], and on 28 June 2023, it published a Directorial Decree n. 309-MinAmbiente [12], integrating article 272-bis, in which the HMA is identified among anthropic activities as having a potential odor impact and whose application for authorization must therefore involve the description and evaluation of odor emissions. Like other European regulations, this guideline is based on dynamic olfactometry and dispersion modeling; however, even though it defines the requirements of the odor impact studies by simulation, it does not set any acceptability criteria.

Currently, environmental monitoring solutions are characterized by high costs and the need for extensive resources. Odor impact is generally determined from concentration data, expressed in odor units per cubic meter, i.e., the number of dilutions necessary so that 50% of human sensory panels no longer perceive the smell of the sample analyzed [13]. This type of analysis presents some limitations, such as high costs of air sampling-laboratory management and, mostly, the impossibility of continuous measurements. Electronic noses could represent the best solution for meeting the expectations for environmental issues regarding odor annoyance. Nevertheless, their use is still limited due to technological problems (e.g., sensor drift, variability due to atmospheric conditions, etc.).

Thus, for the qualitative and/or quantitative determination of VOCs in complex matrices, the technique of choice is represented by hyphenated methods such as gas chromatography (GC) with mass spectrometry (MS). To identify odor characteristics and the intensity of the detected VOCs, an accompanying investigation by GC–Olfactometry (GC–O) is binding [14]. In principle, a GC–O is a sniffing device with a split at the end of the GC column. Trained sensory panelists sniff the eluate—combined with a heated and additionally humidified inert gas, in parallel with MS detection—describing each perceived odor and its relative intensity in parallel to the detection of the substances by GC–MS.

However, it is still difficult to directly inject the sample into a GC system to identify small molecules. So, the sample preparation step, consisting of the extraction, purification, and enrichment of analytes, is primary to obtain accurate results. Recently, thanks to automation developments, mainly due to an increasing demand for Green Analytical Chemistry (GAC) [15], monolithic silica and polymers were modified to suit devices for the extraction and enrichment of analytes in various matrices (environmental, food, and biological). This approach contributed to miniaturization and automation by on-line pre-concentration, which can reduce the time and the cost of sample preparation [16,17,18].

Monolithic material was introduced in 1989 by Hjerten et al. [19]. Its preparation is performed through polymerization of a monomer mixture with a porogen solvent. The Monolithic Material Sorptive Extraction (MMSE) technology offers chemical stability over a wide pH range, with pores in a monolithic structure having a large surface area of at least 150 m^2^/g to allow for a simple method of sample preparation. In 2009 [20,21], a commercial miniaturized monolithic hybrid adsorptive device, called MonoTrap^TM^ (GL Science Inc., Tokyo, Japan) [22]—made of high-purity silica and/or silica with activated carbon, graphite, or chemically modified octadecyl silane (ODS)—was introduced as a sampling device, particularly recommended for polar compounds. More recently, polydimethylsiloxane (PDMS) with graphitic carbon has been used as an additional sorbent phase.

Using vacuum as a pre-equilibration step, the degree of headspace (HS) partitioning for VOCs reducing the pressure increases. In 2001, Brunton et al. [23] reported the positive effects of low-pressure HS–solid phase microextraction (SPME) sampling of food aroma volatiles from raw turkey, while in 2012 Psillakis et al. [24] evacuated air from a sampling container by using a tailor-made closure before introducing a liquid sample, designating the method as vacuum-assisted HS-SPME (Vac-HS-SPME). Currently, custom-designed closures offer gastight seals to commercial 20 mL HS vials, allowing for microextraction sampling under vacuum conditions. If, to date, a few studies have focused on Vac-HS sampling for liquid samples using the SPME technique [25,26], even fewer are proposed for solid samples [27,28].

The challenge faced by this work include the chemical characterization of the odorous emissions from HMA via new, innovative sampling by MonoTrap^TM^. To contribute to the growing use of this technology, this study explores the vial preparation method involved in Vac-HS-MonoTrap^TM^ sampling from solid matrices and the related analysis performed by GC–MS/O. Likewise, the optimization of analytical parameters was performed throughout the application of design of experiments (DoE) that allowed us to carry out fewer experiments for method development.

## 2. Results and Discussion

To date, there are few analytical methods [29,30,31,32] for the determination of the odor-active compounds of HMA; the gap especially occurs for polar VOCs. Vac-HS-MMSE-MonoTrap^TM^ sampling and following GC–MS/O analysis was investigated as a possible alternative to conventional methods for odorous compound determination, to provide a simple, fast, sensitive, and solvent-free innovative procedure for HMA fingerprint. Ultimately, this technique allowed us to distinguish 35 odor-active compounds in HMA. These compounds are represented by aldehydes, alcohols, and ketones, having an OT that decreases markedly to sub-ppb as the number of carbon atoms increases.

Considering what is indicated above, Table 1 shows the results as odorous compounds identified by mass spectrum (compared from the mass spectra library), GC–O analysis, the retention times, and LTPRI (see Section 3.7). The VOC cut-off was based on the retention time of the tridecane (RT 25.8 min), a C_13_ *n*-alkane. All the odor-active compounds considered have a BP and VP lower than those of tridecane—232 °C and 10 Pa, respectively—except for 1-decanol (1 Pa), due to its low (0.7 ppb) odor threshold (OT).

The authors satisfied three fundamental requirements for the use of MonoTrap^TM^ as diffusive sampler—the high sampling rate, the most suitable adsorbent phase, and the effectiveness of the vacuum-effect (by heat transfer theory, chemometric approach, and the theory of ideal gas, respectively)—to achieve adequate performances.

**Figure 1:**
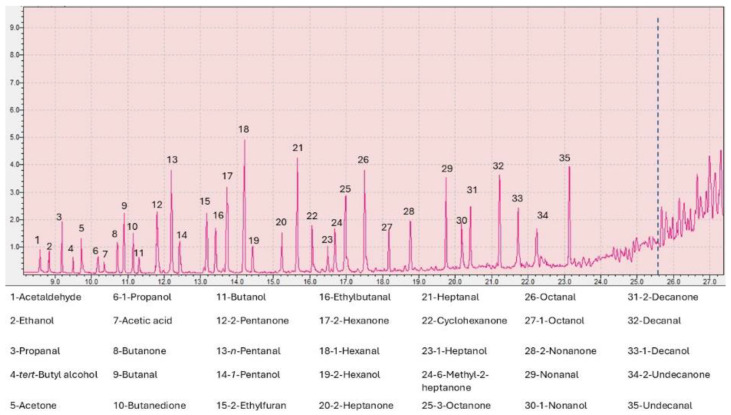
Chromatogram of the odorous compounds identified by GC-MS/O analysis and LTPRI. At the RT of 25.8 min—corresponding to tridecane—the cut-off marks the VOCs considered, as indicated by the blue dotted line.

**Figure 2:**
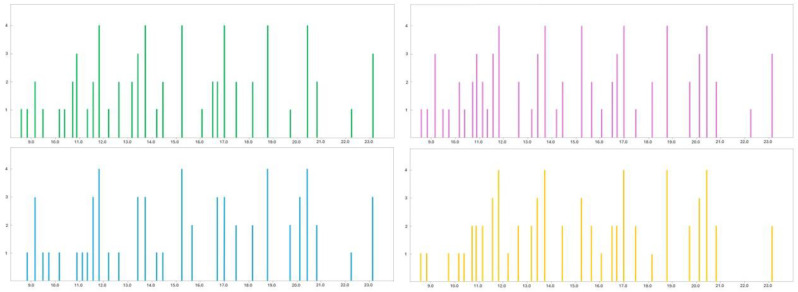
Elaboration of the GC-O analysis of the four panelists; the data show the olfactory detections observed by each panelist (represented by different lines for each odor perceived) during the elution of the samples. Different colors are associated with different panelists.

Figure 1 reports the chromatogram obtained with the developed method, whose optimization is described in detail in the following paragraphs; in Figure 2 the data of the olfactory analysis carried out by the four panelists are shown. The data for each panelist were obtained by subsequent repetitions of different aliquots of HMA sample.

The strong, often acrid, pungent, and penetrating odor of the compounds detected can be traced back to that released by HMA. In particular, saturated fatty aldehydes with a molecular weight (MW) below 150 Da exhibit unpleasant aromas, while higher MW aldehydes can exhibit sweet and fruity aromas [36,37,38,39]. Conversely, alcohols above C_9_ have fattier and more unpleasant oily odor notes [37], differently from most straight-chain C_4_–C_9_ alcohols with a fruit-like aroma.

### 2.1. Heat Transfer Theory

The MonoTrap^TM^ technology was selected based on its capacity to load the highest mass of analytes. The adsorption processes of solid, porous phases occur on the sorbent surface; the substantial thickness of the coating allows the analyte to be retained exclusively within the pores of the solid phase. This theoretical framework can be effectively employed to minimize the number of experiments required to predict trends in MonoTrap^TM^ analysis; however, the assumption of ideal conditions necessary for mathematical modeling should be verified. To calculate n, the mass (ng) of the adsorbed analyte in a sampling time t (s), using a porous coating, the theory of heat transfer can be applied [40,41,42]:

(1) $$n=\frac{D_{g}\times A}{\delta }\times C_{g}\times t$$

where D_g_ represents the diffusion coefficient in air (cm^2^ s^−1^), *A* the surface of the sorbent phase (i.e., 2.67 cm^2^), C_g_ the concentration of the analyte in the 20 mL vial (0.5 ng mL^−1^), and δ the thickness of the boundary layer surrounding the MonoTrap^TM^ (cm), defined as follows:

(2) $$\delta =9.52\times \frac{b}{{R_{e}}^{0.62}\times {{\;S}_{c}}^{0.38}}$$

with the Reynolds number (Re) expressed as 2ubv^−1^, where u is the linear air speed (cm s^−1^), v is the air viscosity (0.014607 cm^2^ s^−1^), b is the radius of the MonoTrap^TM^ (0.19 cm), and Schmidt’s number (Sc) is defined by vD_g_^−1^. In theory, linear air speeds exceeding 10 cm s⁻^1^ result in a *δ* value approaching zero, thereby rendering Equation (1) invalid. By means of Equation (1), we calculated the theoretical uptake (ng s^−1^) and the theoretical SR (mL min^−1^) for each of the 35 substances surveyed.

Table 2 presents the *D_g_*, the theoretical uptakes and theoretical SRs for each substance, both at atmospheric pressure and in vacuum condition. Conversely, the SRs at 11.6 mbar are significantly enhanced, by a factor of approximately more than two orders of magnitude. This result indicates that the performance of MonoTrap^TM^ improves, suggesting that the signal enhancement observed when working at reduced pressure is likely due to an enhanced sorbent capacity, in addition to a more efficient stripping from the matrix.

### 2.2. Choice of the MonoTrap^TM^ Adsorbent Phase Through a Chemometric Approach

The automation with a three-axis autosampler on-line with the GC allowed for a high-throughput analytical procedure, and it also delivered the ability to define the best conditions for the development of the method regarding the choice of the adsorbent phase to adopt.

**Figure 3:**
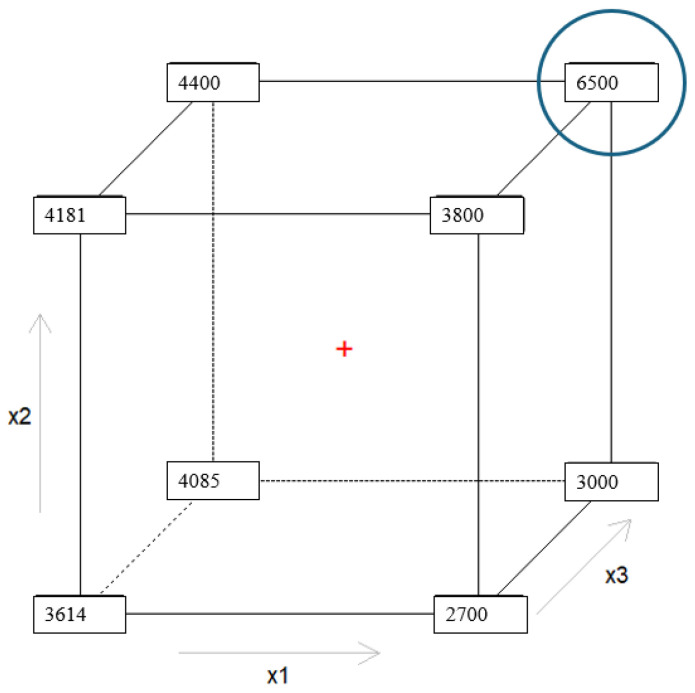
Experimental domain reporting the areas under the peak area intensities obtained for the eight experiments for the 1-hexanal. In blue, experiment number eight is circled, which allows one to obtain the highest sensitivity.

The optimization of the extraction procedure on HMA, conducted by applying the 2^3^ full factorial design [43,44,45], was performed by investigating three main variables: the RGPSTD and RGC18-TD MonoTrap^TM^ (x_1_), the extraction step conducted under vacuum (x_2_), and the equilibration step (x_3_). From preliminary studies, it was decided to exclude the use of RSC18-TD from the experimental matrix because it was able to extract less analytes from the matrix compared to the other adsorbent phases, and it could not study equilibration times like 12 min due to the decrease in sensitivity. These parameters were optimized to maximize the peak area intensities (y_1_, y_2_, y_3_, and y_4_) of four compounds (butanal, 1-hexanal, heptanal, decanal, respectively), performing only eight experiments. The results highlighted that the models computed for y_2_, y_3_, and y_4_ were optimized when the three variables (x_1_, x_2_, and x_3_) were in the high level (equal to 1), as shown in Figure 3. This implies that the MonoTrap^TM^ used to perform the extraction must be the RGC18-TD (x_1_ = 1), the Vac extraction must be employed (x_2_ = 1), and lastly the equilibration step must last 4 min (x_3_ = 1).

**Figure 4:**
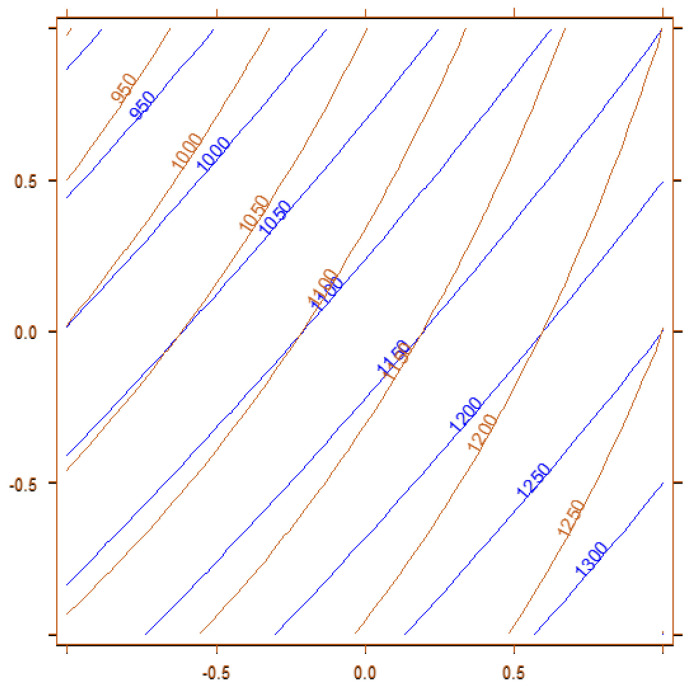
Overlapping of the contour plots obtained for the model describing y_1_, peak areas of butanal. Blue lines describe the variables x_1_ vs. x_2_, and red lines describe the variable x_1_ vs. x_3_.

Instead, the model for y_1_, which describes the sensitivity for butanal, has a different behavior compared to the other molecules, showing higher responses with the extraction performed at atmospheric pressure (x_2_ = −1) and the equilibration time set at 8 min (x_3_ = −1); see Figure 4 for further details.

**Figure 5:**
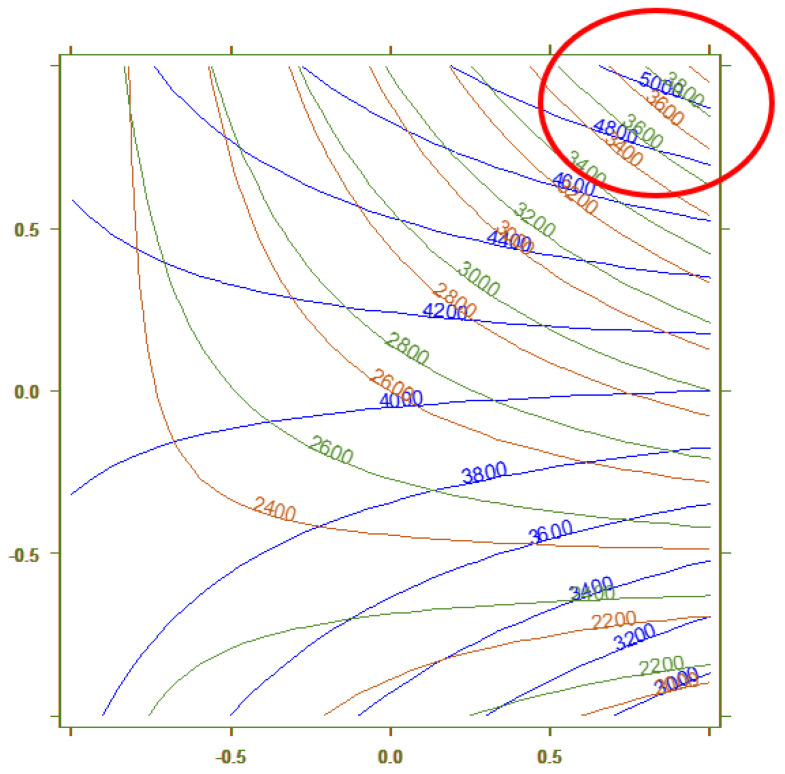
Overlapping of the contour plots obtained, computing variables x_1_ vs. x_2_ for the models of y_2_ (blue lines), y_3_ (red lines), and y_4_ (green lines).

Thus, the experimental conditions selected to simultaneously determine the presence of VOCs at cut-off in HMA are those reported in Table 3 (experiment number eight) and highlighted in Figure 4, which optimize the signals for most of the analytes assessed. Finally, comparing Figure 4 and Figure 5, it is understandable that the authors chose to select the optimal experimental conditions as x_1_ = 1, x_2_ = 1, and x_3_ = 1 (top right corner of Figure 3 and Figure 5) instead of x_1_ = 1, x_2_ = −1, x_3_ = −1 (bottom right corner of same figures): the decrease in sensitivity for y_1_ is lower compared to the one that other analytes would have.

### 2.3. Vacuum Effect on HS-MMSE-MonoTrap^TM^ Sampling

The research conducted on HMA by Vac-HS-MMSE-MonoTrap™ demonstrated that the utilization of a higher phase volume and layer thickness enables the successful coupling of sample heating at elevated temperatures (160 °C) and vacuum sampling. This approach reduces the equilibrium time, thereby maximizing VOC extraction, particularly for those with a higher molecular weight. This outcome aligns with the observations made at lower temperatures in other matrices [46]. In vacuum conditions, elevated sampling temperatures were demonstrated to result in a reduction in extraction efficiency compared to atmospheric pressure. This phenomenon was found to be associated with increased humidity levels occurring during the heating of the sample, with a more pronounced effect observed when an absorbent type (such as PDMS) is used [47]. Indeed, extraction efficiencies obtained under vacuum and at a mild sampling temperature are comparable with those at regular pressure and a much higher sample temperature; this is coherent with the general decrease in boiling point, observable in vacuum conditions, for organic compounds [40,48,49,50]. Lastly, elevated temperatures might reduce the effect of vacuum; the VP of analytes and volatile matrix components increases exponentially, therefore enhancing the total pressure inside the sample container. Following the theory of ideal gas, in an empty 20 mL HS crimp-top vial, evacuated at an absolute pressure of 8 mbar and then heated to 160 °C, the pressure increases up to 11.6 mbar. This condition can be extended even when the 20 mL HS crimp-top vial is loaded with a small amount of dry solid sample which also does not release large amounts of VOCs, since in the presence of water or other volatile compounds, the maximum vacuum that can be achieved depends on the liquid–vapor equilibrium in the phase diagram.

The analytes present within solid samples (adsorbed, dissolved, and/or in gaseous phases) result in greater resistance to volatilization compared with liquid samples [51]. In a modified form of Fick’s law of diffusion, Yiantzi et al. [52] stated that a reduction in total pressure would result in a vapor flux increase at the solid surface, thereby accelerating the volatilization rate and shifting the equilibrium towards a higher analyte concentration in the HS. We found that the operational fundamentals of the sample for an ideal workflow using 20 mL HS vials included evacuation at 8 mbar for 10 s.

The greater response produced by volatiles was revealed in HMA 1-hexanal, heptanal, octanal, nonanal, and decanal using Vac assistance, while propanal and butanal with no-Vac. A comparison of performance, denoted as Vac/no-Vac ratios, can be calculated by dividing the compound response from Vac-HS-MMSE-MonoTrap^TM^ sampling by the compound response from HS-MMSE-MonoTrap^TM^ without vacuum assistance (Vac/no-Vac ratio), which resulting in the following values: hexanal 4.1, 3-octanone 7.7, octanal 8.1, 1-octanol 8.8, 2-nonanone 9.1, nonanal 9.2, 1-nonanol 9.8, and decanal 10.9. As the BP of target analytes increased, the Vac/no-Vac response ratios also generally increased in agreement with Solomou et al. [53]. When Vac is removed, there is poor response for 2-decanone, 2-undecanone, and undecanal.

## 3. Materials and Methods

### 3.1. Reagents

The *n*-alkanes butane (99%, CAS n. 106-97-8), pentane (99%, CAS n. 109-66-0), hexane (>95%, CAS n. 110-54-3), heptane (99%, CAS n. 142-82-5), octane (>99%, CAS n. 111-65-9), nonane (>99%, CAS n. 111-84-2), decane (99%, CAS n. 124-18-5), undecane (>99%, CAS n. 1120-21-4), dodecane (99%, CAS n. 112-40-3), tridecane (>99%, CAS n. 629-50-5), and the internal standard 2,4,6-trimethylpyridine (99%, CAS n. 108-75-8) were purchased from Supelco (Merck KGaA, Darmstadt, Germany) and used for evaluation of the (*i*) LTPRI and (*ii*) VOC cut-off based on the RT of the tridecane.

### 3.2. Vac-HS-MMSE-Monotrap^TM^ TD Procedure

A Kit Vac-SPME-Fiber (part no. 20-102) by ExtraTECH Analytical Solutions equipped with a conditioned/ready-to-use cylindrical Thermogreen^TM^ LB-2 septa (part no. 20608, Supelco) was purchased from Markes International Ltd. (Bridgend, UK) and used for all experiments for closure of the 20 mL HS crimp-top vial (Markes International Ltd.). A LABOPORT^®^ N820G (KNF Service GmbH, Freiburg im Breisgau, Germany) pumping unit (8 mbar ultimate vacuum without gas ballast) connected to an 18-gauge (1.219 mm external diameter) needle was used to evacuate the air inside the 20 mL HS crimp-top vial. The needle was also used to support the MonoTrap^TM^.

Disposable, ready-to-use, and preconditioned MonoTrap^TM^ TD rods (external diameter 2.9 mm, internal diameter 1 mm, length 10 mm) were purchased from GL Sciences (Shinjuku, Tokyo, Japan) in ampoules for single-use: (*i*) RSC18-TD (part no. 1050-73201) as silica gel with ODS as the functional group for hydrophobic analytes with medium (250 °C) to high (300 °C) BP, (*ii*) RGC18-TD (part no. 1050-74201) as graphite carbon with ODS for polar or hydrophobic analytes with low (200 °C) to medium BP, and (*iii*) RGPS-TD (part no. 1050-74202) as graphite carbon with PDMS for polar or hydrophobic analytes with a low to medium BP.

### 3.3. Sample and Sampling

The HMA analyzed was the conventional Surface Layer (SL) made (*w*/*w*) of 5% bitumen (50/70 type), 65% aggregate, 8% filler, and 22% Reclaimed Asphalt Pavement (RAP). The relative humidity (RH) of the HMA-SL declared by the manufacturer was less than 1%. Two grams of HMA-SL samples and MonoTrap^TM^ rod were loaded into a 20 mL HS crimp-top vial before pulling a vacuum. Then, the vial was heated at the bottom at 160 °C and cooled at the head with compressed air at 25 °C. After adsorption, the MonoTrap^TM^ rod was placed in a liner and desorbed.

### 3.4. Three-Axis Autosampler and Multi-Mode GC Inlet Systems

A three-axis Shimadzu AOC-6000 Plus Multifunctional Autosampler (Shimadzu, Kyoto, Japan) was used, on-line with GC for a fully automated analysis. After the sampling, the MonoTrap^TM^ containing the collected analytes was placed in a GC liner for thermal desorption and sealed with the Capping-De-Capping (CDC) station (GL Sciences) moved by the Automatic LINer EXchanger (LINEX, GL Sciences); both are accessories of the OPTIC-4 (GL Science) multi-mode GC inlet system. The analytes adsorbed on MonoTrap^TM^ were desorbed into the GC–MS directly into the CryoFocus-4 (GL Sciences), a cryo-trap at the head of the GC column cooled by liquid nitrogen to sub-ambient temperature (−150 °C for 300 s). After trapping, the analytes were released from the cryo-trap using a fast heating (60 °C s^−1^ up to 290 °C), ensuring that they were introduced onto the capillary column in a very sharp band.

### 3.5. GC–MS/O

The GC instrument used was a Shimadzu GC-2030 with QP2020 NX (Shimadzu) MS detector. To select olfactometrically detected analytes from volatile analytes, a sniffing port PHASER Pro (GL Sciences) with Olfactory Voicegram and a GC–O Aroma Palette was configured in-line to GC–MS by a transfer-line. The sample was split 1:20 *v*/*v*; the effluent from the capillary column was equally split between the detection systems A J&W GC column VF-5ms (part no. CP8949, length 60 m × internal diameter 0.25 mm × film thickness 1 μm) provided by Agilent Inc. (Santa Clara, CA, USA) was used. Helium was used as carrier gas at 0.9 mL min^−1^. The oven temperature program was 45 °C (5 min hold) to 320 °C (10 °C min^−1^), with a final hold of 8 min. MS conditions were as follows: detector interface temperature 250 °C, ion source temperature 230 °C, ionization energy 70 eV, and mass range 28.5–300 amu. Based on the chromatogram data obtained from GC–MS, we used the National Institute of Standards and Technology 11 Mass Spectral Library and Flavor & Fragrance Natural & Synthetic Compounds GC–MS library (both from Shimadzu) for searching for mass spectra with a similarity score of 90% or higher to identify the analytes.

A panel of four sensory panelists (2 men and 2 women, aged from 25 to 34) from the laboratory staff with previous GC–O sniffing experience was assembled in the GC–MS/O study. When an aroma was being detected, the sensory panelists were asked to press a specific button on the instrument keypad to record the time, the description, and odor intensity (noted by a voice recording system). The scale used for intensity was 1–4 (1 = weak, 2 = moderate, 3 = strong, 4 = very strong). The analysis followed the guidelines of Pollien et al. [54]. Each sample was evaluated consecutively by each of the sensory panelists.

**Figure 6:**
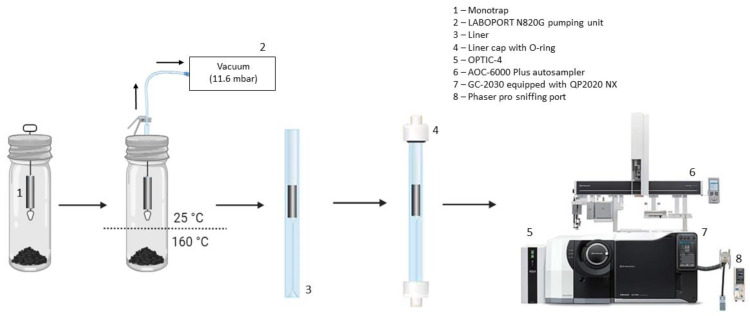
Flow chart and image of xyz-autosampler for the automated Vac-HS-MonoTrap^TM^ sampling on-line with GC-MS/O instrumentation.

Figure 6 reports the scheme of the GC-MS instrument, equipped with the olfactory port, as well as a brief flow chart of the sampling process.

### 3.6. Chemometric Tool

Microsoft Excel (version 18.0) was used to collect data, while an open source and R-based software (version 4.3.3), Chemometric Agile Tool (CAT) [55], was used to process it.

First, a 2^3^ full factorial design was applied to optimize the analytical method developed on four compounds considered (butanal, 1-hexanal, heptanal, decanal), which means that three factors were studied at two levels each. The first factor (x_1_) considered the use of RGPS-TD (low level, −1) and RGC18-TD (high level, 1) MonoTrap^TM^ in the extraction step. The second one (x_2_) explained the use of Vac during the extraction step, low level for the extraction at atmospheric pressure and high level for the extraction under Vac. And last, the third factor (x_3_) referred to the duration of the equilibration step, which was low level for 8 min and high level for 4 min.

### 3.7. Identification of VOCs by LTPRI

An important tool for the identification of compounds is the use of Retention Indexes that were developed originally by Kovats [56] for isothermal analysis and modified by van den Dool and Kratz [57] for linear temperature-programmed analysis. The most used is the latter, named the Linear Temperature-Programmed Retention Index (LTPRI) [58,59,60,61,62]. The LTPRI was defined under identical gas chromatographic conditions of the sample as follows:

(3) $$\mathrm{LTPRI}=100\times \frac{t_{R(A)}-t_{R(C)}}{t_{R(C+1)}-t_{R(C)}}+100\times C$$

where t_R(A)_ is the analyte retention time, t_R(c)_ is the retention time of the n-alkane eluting immediately before the analyte, t_R(C+1)_ is the retention time of the n-alkane eluting immediately after the analyte, and C is the number of carbon atoms for t_R(C)_.

Volatile compounds were identified by matching the mass spectra to the database and the LTPRI of each compound with its reference values.

## 4. Conclusions

The introduction of miniaturized analytical solutions in recent years is noteworthy and consistent with the needs of Green Analytical Chemistry, a virtuous trend of continuous improvement in the framework of an increasing preservation of the environment. Several miniaturized techniques are currently available on the market and integrated with new analytical solutions, such as MonoTrap^TM^ coupled with vacuum-assisted extraction.

As for the monitoring of VOC emissions from anthropic sources, i.e., HMA from industrial plans, the Vac-HS-MonoTrap^TM^ with the GC–MS/O analytical approach has proved to be successful. The main advantages reside in a larger surface area, a high sensitivity, a high uptake, especially for the polar VOCs, and no need for derivatization steps. Moreover, reducing the pressure in the sample vial as a pre-equilibration step increases the degree of HS partitioning for dry solid samples. A chemometric approach was used to optimize the method with as few experiments as possible. The developed method, tested on real HMA samples, allowed for the generation of an emission fingerprint, represented by an MS chromatogram and a matching odorgram.

The main compounds associated with the HMA odor fingerprint result in aldehydes, ketones, and alcohols. Alcohols do not show evidence of toxic activity on reproductive systems or developing organisms, but their inhalation could provoke irritation. Although the potential risks associated with aldehyde and ketone exposure are well documented, the toxic mechanisms remain poorly understood.

The toxicological implications will require careful quantification of the compounds present in the emission, broadening the panorama regarding the odor component investigated here.

The combination of analytical chemistry, engineering, and biomedical science has enabled significant advances in the understanding of odorous emissions. Future progress has the potential to safeguard public health and environmental well-being while simultaneously supporting the achievement of sustainable development goals.

## Figures and Tables

**Table 1:** Olfactometrically detected VOCs in HMA by Vac-HS-MonoTrapTM sampling and GC-MS/O analysis.

Num.	Compound ** (Name/ Formula)	CAS n.	MW Da	BP ^a,d,e^ °C	VP ^a,d,e^ Pa	RVD *^,a^ Air = 1	LTPRI ^b^ Estimated	Retention Times (RTs)	Peak Area Score Units ^f^	Odor Smell	OT ^c^ ppb
1	Acetaldehyde/C_2_H_4_O	75-07-0	44	20	101,000	1.5	412	8.342	+	Pungent, fruity	1.5
2	Ethanol/C_2_H_6_O	64-17-5	46	78	5800	1.6	458	8.501	+	Weak	520
3	Propanal/C_3_H_6_O	123-38-6	58	49	31,000	2.0	471	9.117	+++	Pungent, choking	1.0
*4*	*tert*-Butyl alcohol/C_4_H_10_O	75-65-0	74	83	4100	2.6	476	9.304	+	Camphorous	4500
5	Acetone/C_3_H_6_O	67-64-1	58	56	24,000	2.0	478	9.398	+	Fruity	42,000
6	1-Propanol/C_3_H_8_O	71-23-8	60	97	2000	2.1	533	10.087	+	Weak	94
7	Acetic acid/C_2_H_4_O_2_	64-19-7	60	118	1500	2.1	543	10.228	+	Strong, vinegar-like	6
8	2-Butanone/C_4_H_8_O	78-93-3	72	79	10,500	2.41	548	10.431	++	Mint	440
9	Butanal/C_4_H_8_O	123-72-8	72	75	12,200	2.5	556	10.535	+++	Pungent	0.6
10	Butanedione/C_4_H_6_O_2_	431-03-8	86	88	7600	3.0	567	11.079	++	Chlorine-like	0.05
11	Butanol/C_4_H_10_O	71-36-3	74	117	580	2.6	607	11.187	+	Harsh	38
12	2-Pentanone/C_5_H_10_O	107-87-9	86	101	1600	3.0	647	11.328	+++	Aceton-like	28
13	*n*-Pentanal/C_5_H_10_O	110-62-3	86	103	3400	3.0	664	11.488	++++	Acrid, pungent	0.41
14	1-Pentanol/C_5_H_12_O	71-41-0	88	138	600	3.0	742	12.136	+	Fusel-like	100
15	2-Ethylfuran/C_6_H_8_O	3208-16-0	96	92	6666	-	756	12.375	++	Smoky burn	-
16	2-Ethylbutanal/C_6_H_12_O	97-96-1	100	116	2000	-	762	13.108	++	Pungent	-
17	2-Hexanone/C_6_H_12_O	591-78-6	100	126	360	3.5	770	13.247	+++	Sharp	24
18	1-Hexanal/C_6_H_12_O	66-25-1	100	129	1100	-	780	13.378	++++	Strong, green grass	0.28
19	2-Hexanol/C_6_H_14_O	626-93-7	102	136	2300	3.5	795	14.087	+	Sweet	6
20	2-Heptanone/C_7_H_14_O	110-43-0	114	151	200	3.9	859	14.252	++	Penetrating-spicy	6.8
21	Heptanal/C_7_H_14_O	111-71-7	114	153	3500	-	878	15.143	++++	Pungent, fatty	0.18
22	Cyclohexanone/C_6_H_10_O	108-94-1	98	156	500	3.4	891	15.369	++	Peppermint-like	-
23	1-Heptanol/C_7_H_16_O	111-70-6	116	175	15	4.01	920	16.065	+	Aromatic	4.8
24	6-Methyl-2-heptanone/C_8_H_16_O	928-68-7	128	168	173	-	941	16.297	++	Camphorous	-
25	3-Octanone/C_8_H_16_O	106-68-3	128	168	503	-	951	16.386	+++	Sharp, mild fruit	-
26	Octanal/C_8_H_16_O	124-13-0	128	171	206	-	965	16.584	++++	Pungent citrus-like	0.01
27	1-Octanol/C_8_H_18_O	111-87-5	130	194	10	4.5	984	17.308	++	Strong, aromatic	2.7
28	2-Nonanone/C_9_H_18_O	821-55-6	142	192	63	-	1038	18.131	++	Herbaceous	-
29	Nonanal/C_9_H_18_O	124-19-6	142	195	37	-	1061	18.465	++++	Orange–rose	0.34
30	1-Nonanol/C_9_H_20_O	143-08-8	144	213	10	-	1078	19.448	++	Citronella oil-like	0.9
31	2-Decanone/C_10_H_20_O	693-54-9	156	210	25	-	1146	20.092	+++	Orange, fatty peach	-
32	Decanal/C_10_H_20_O	112-31-2	156	212	10	-	1167	20.241	++++	Penetrating waxy	0.4
33	1-Decanol/C_10_H_22_O	112-30-1	158	230	1	5.5	1254	20.462	++	Fruity	0.7
34	2-Undecanone/C_11_H_22_O	112-12-9	170	231	10	-	1268	22.138	+	Strong	-
35	Undecanal/C_11_H_22_O	112-44-7	170	223	10	-	1276	23.087	+++	Penetrating orange	-

* Relative vapor density (RVD) ^a^. ** Identification: MS, mass spectra (identified from the mass spectra deposited in a database) and Linear Temperature-Programmed Retention Index (LTPRI) (compared with the LTPRI in the literature). ^a^ International Labour Organization (ILO) [33]. ^b^ Performed by the author of this work as Materials and Method Section 3.5: J&W GC column VF-5ms column (length 60 m × internal diameter 0.25 mm × film thickness 1 μm). ^c^ Yoshio Nagata. Measurement of Odor Threshold by Triangle Odor Bag Method [34]. ^d^ PubChem—open chemistry database at the National Institutes of Health (NIH). ^e^ ChemSpider—Royal Society of Chemistry [35]. ^f^ The data are the average values of the odor perception (expressed from “+”, weak, to “++++”, very strong) obtained by the olfactory analysis of the four panelists.

**Table 2:** Diffusion coefficient ^a,b^, theoretical uptake and theoretical sampling rate (SR) for each substance surveyed, calculated at atmospheric pressure and in vacuum conditions (i.e., 11.6 mbar).

Num.	Compound Name	Atmospheric Pressure			Vacuum		
		*D_g_* cm^2^/s	Uptake ng/s	SR mL/min	*D_g_* cm^2^/s	Uptake ng/s	SR mL/min
1	Acetaldehyde	0.13	0.08	9.10	12.10	1.26	151
2	Ethanol	0.12	0.07	8.66	11.40	1.21	146
3	Propanal	0.11	0.07	8.20	10.00	1.12	134
*4*	*tert*-Butyl alcohol	0.091	0.06	7.29	8.40	1.01	121
5	Acetone	0.11	0.07	8.20	10.00	1.12	134
6	1-Propanol	0.1	0.06	7.73	9.50	1.08	130
7	Acetic acid	0.11	0.07	8.20	10.40	1.15	138
8	2-Butanone	0.094	0.06	7.44	8.70	1.03	123
9	Butanal	0.094	0.06	7.44	8.70	1.03	123
10	Butanedione	0.092	0.06	7.34	8.40	1.01	121
11	Butanol	0.091	0.06	7.29	8.30	1.00	120
12	2-Pentanone	0.084	0.06	6.94	7.70	0.95	114
*13*	*n*-Pentanal	0.084	0.06	6.94	7.70	0.95	114
14	1-Pentanol	0.082	0.06	6.84	7.50	0.94	112
15	2-Ethylfuran	0.08	0.06	6.73	7.40	0.93	112
16	2-Ethylbutanal	0.077	0.05	6.58	7.10	0.91	109
17	2-Hexanone	0.077	0.05	6.58	7.00	0.90	108
18	1-Hexanal	0.077	0.05	6.58	7.00	0.90	108
19	2-Hexanol	0.075	0.05	6.47	6.90	0.89	107
20	2-Heptanone	0.071	0.05	6.25	6.50	0.86	103
21	Heptanal	0.071	0.05	6.25	6.50	0.86	103
22	Cyclohexanone	0.078	0.06	6.63	7.20	0.91	110
23	1-Heptanol	0.069	0.05	6.14	6.30	0.84	101
24	6-Methyl-2-heptanone	0.066	0.05	5.98	6.10	0.82	98.9
25	3-Octanone	0.066	0.05	5.98	6.10	0.82	98.9
26	Octanal	0.066	0.05	5.98	6.10	0.82	98.9
27	1-Octanol	0.065	0.05	5.92	5.90	0.81	96.9
28	2-Nonanone	0.062	0.05	5.75	5.70	0.79	94.9
29	Nonanal	0.062	0.05	5.75	5.70	0.79	94.9
30	1-Nonanol	0.061	0.05	5.69	5.60	0.78	93.8
31	2-Decanone	0.058	0.05	5.52	5.40	0.76	91.7
32	Decanal	0.058	0.05	5.52	5.40	0.76	91.7
33	1-Decanol	0.058	0.05	5.52	5.30	0.76	90.7
34	2-Undecanone	0.056	0.04	5.40	5.10	0.74	88.5
35	Undecanal	0.056	0.04	5.40	5.10	0.74	88.5

^a^ Advamacs—TriMen Chemicals (Łodz, Poland). ^b^ U.S. Environmental Protection Agency (EPA)—EPA On-line Tools for Site Assessment Calculation.

**Table 3:** Description of the experiments performed to optimize the analytical method by DoE.

	Experimental Matrix			Experimental Plan		
Exp	x_1_	x_2_	x_3_	MonoTrap^TM^	Vac	Equilibration min
1	−1	−1	−1	RGPS TD	No	8
2	1	−1	−1	RGC18 TD	No	8
3	−1	1	−1	RGPS TD	Yes	8
4	1	1	−1	RGC18 TD	Yes	8
5	−1	−1	1	RGPS TD	No	4
6	1	−1	1	RGC18 TD	No	4
7	−1	1	1	RGPS TD	Yes	4
8	1	1	1	RGC18 TD	Yes	4

## Data Availability

Data will be made available on request.
